# 

**Published:** 1888-10

**Authors:** 


### The Independent Practitioner—Vol. IX.

### PUBLISHED BY DENTISTS FOR DENTISTS.

### PROSPECTUS.

The Independent Practitioner has assumed a more important role as the organ of the International Dental Journal Company. The promises of the New York Dental Journal Association of making Volume IX better than any former volumes have been well carried out. The scope of the Journal has been enlarged, and its circle of influence greatly increased. We still continue the magnificent offer of Dr. Stowell's work on the *Microscopic Structure of a Human Tooth*, which is published at the moderate price of six dollars each. We have made such arrangements with the publishers that we can furnish it in connection with a subscription to **The Independent Practitioner for One Dollar and Fifty Cents. That is, to every one who will send in Four Dollars we will send the Independent Practitioner one year and a copy of this beautiful and useful atlas.**

This offer is intended only for subscribers in America. Foreign residents within the postal union must enclose fifty cents extra for prepayment of postage upon the Journal. Our regular rate of subscription to such is three dollars per annum.

As the book absolutely costs more than the sum for which it will be furnished, the money must accompany the subscription. Nor will the book be sold separately. It will only be sent in connection with a subscription to this journal.

The book will be sent either by mail or express. If it is desired that it should be sent by mail, the postage (twenty-five cents) should be sent. All remittances should be sent to the Editor and Business Manager, Dr. W. X. Sudduth, 1215 Filbert Street, Philadelphia, Pa. Postal orders, Postal Notes, or New York Drafts are the safest and most convenient.

The Independent Practitioner has been transferred to a new and enlarged syndicate—no material change is, however, to be made in the form of the journal until the close of the present volume. We hope to hold all our old subscribers, and obtain many new ones. You may hand your subscription to any one of the stockholders, a list of whose names is here appended.

GEO. S. ALLAN,
R. R. ANDREWS,
H. A. BAKER,
W. C. BARRETT,
A. P. BEALE,
S. T. BEALE,
C. S. BECK,
G. V. BLACK,
E. A. BOGUE,
C. F. W. BODECKER,
W. G. A. BONWILL,
EDWARD C. BRIGGS,
THOS. BRADLEY,
ALBERT H. BROCKWAY,
A. E. BROWN,
WM. CARR,
G. F. CHENEY,
DWIGHT M. CLAPP,
JOHN D. CODMAN,
CHAS. D. COOK,
J. E. CRAVENS,
J. N. CROUSE,
EDW. T. DARBY,
D. M. DAVIDSON,
WM. H. DWINELLE,
CHAS. J. ESSIG,
J. N. FARRAR,
T. FILLEBROWN,
W. B. FINNEY,
T. A. FLETCHER,
M. W. FOSTER,
CHAS. E. FRANCIS,
W. F. FUNDENBERG,
W. H. FUNDENBERG,
E. C. FUNDENBERG,
A. H. GILSON,
E. S. GAYLORD,
J. P. GERAN,
S. H. GUILFORD,
JOHN J. HART,
O. E. HILL,
J. F. P. HODSON,
J. MORGAN HOWE,
ROBERT HUEY,
LOUIS JACK,
G. L. S. JAMESON,
C. A. KINGSBURY,
EDW. C. KIRK,
C. R. E. KOCH,
W. T. LA ROCHE,
W. K. LEECH,
WILBUR F. LITCH,
J. BOND LITTIG,
BENJAMIN LORD,
GEO. W. LOVEJOY, (Canada),
JOHN S. MARSHALL,
JAMES McMANUS,
CHARLES McMANUS,
E. V. McLEOD,
H. J. McKELLOPS,
DAN'L. NEAL McQUILLAN,
HORATIO C. MERIAM,
W. D. MILLER, (Berlin),
W. E. PAGE,
S. B. PALMER,
C. B. PARKER,
D. D. PEABODY,
S. G. PERRY,
C. N. PEIRCE,
CHAS. E. PIKE,
W. A. PHREANER,
W. E. PINKHAM,
H. C. REGISTER,
L. D. SHEPARD,
EUGENE H. SMITH,
B. HOLLY SMITH,
S. G. STEVENS,
W. X. SUDDUTH,
AMBLER TEES,
J. D. THOMAS,
JAMES TRUMAN,
WM. H. TRUEMAN,
F. F. VAN WOERT,
W. W. WALKER,
I. T. WARDWELL,
C. S. WARDWELL,
G. W. WARREN,
L. S. WATERS,
GEO. W. WELD,
JULIUS G. W. WERNER,
JACOB L. WILLIAMS,
R. B. WINDER,
C. A. WOODWARD,
J. A. WOODWARD.

